# Investigation of the Quasi-Brittle Failure of Alashan Granite Viewed from Laboratory Experiments and Grain-Based Discrete Element Modeling

**DOI:** 10.3390/ma10070835

**Published:** 2017-07-21

**Authors:** Jian Zhou, Luqing Zhang, Duoxing Yang, Anika Braun, Zhenhua Han

**Affiliations:** 1 Key Laboratory of Shale Gas and Geoengineering, Institute of Geology and Geophysics, Chinese Academy of Science, Beijing 100029, China; zhoujian@mail.iggcas.ac.cn (J.Z.); anika.braun@rwth-aachen.de (A.B.); hanzhenhua13@mails.ucas.ac.cn (Z.H.); 2 China Earthquake Administration, Institute of Crustal Dynamics, Beijing 100085, China; yangdx@mail.iggcas.ac.cn; 3 College of Earth Science, University of Chinese Academy of Sciences, Beijing 100049, China

**Keywords:** grain-based approach, petrographic texture, grain scale effect, laboratory experiment, Particle Flow Code, Alashan granite

## Abstract

Granite is a typical crystalline material, often used as a building material, but also a candidate host rock for the repository of high-level radioactive waste. The petrographic texture—including mineral constituents, grain shape, size, and distribution—controls the fracture initiation, propagation, and coalescence within granitic rocks. In this paper, experimental laboratory tests and numerical simulations of a grain-based approach in two-dimensional Particle Flow Code (PFC2D) were conducted on the mechanical strength and failure behavior of Alashan granite, in which the grain-like structure of granitic rock was considered. The microparameters for simulating Alashan granite were calibrated based on real laboratory strength values and strain-stress curves. The unconfined uniaxial compressive test and Brazilian indirect tensile test were performed using a grain-based approach to examine and discuss the influence of mineral grain size and distribution on the strength and patterns of microcracks in granitic rocks. The results show it is possible to reproduce the uniaxial compressive strength (UCS) and uniaxial tensile strength (UTS) of Alashan granite using the grain-based approach in PFC2D, and the average mineral size has a positive relationship with the UCS and UTS. During the modeling, most of the generated microcracks were tensile cracks. Moreover, the ratio of the different types of generated microcracks is related to the average grain size. When the average grain size in numerical models is increased, the ratio of the number of intragrain tensile cracks to the number of intergrain tensile cracks increases, and the UCS of rock samples also increases with this ratio. However, the variation in grain size distribution does not have a significant influence on the likelihood of generated microcracks.

## 1. Introduction

The use of granite as a structural engineering material is continuously increasing because of its excellent mechanical properties and resistance to weathering and other environmental influences. Granite is also recognized as an ideal storage material for high-level radioactive waste [1] and as a geothermal reservoir for heat extraction [2,3,4]. The mechanical properties of granitic rock and fracture development are important for a variety of engineering applications such as tunneling, mining, quarrying operations, and slope stability. Quantitative petrographic analysis shows that granitic rock, at the grain scale, shows mineral aggregation with microcracks, which results in a complex inherent microstructure [5,6,7]. Previous laboratory studies of the relationships between the various mechanical properties of granitic rock indicated that mechanical strength and fracture development are related to the inherent microstructure parameters, including mineral composition and distribution, mineral shape, mineral grain size, and pre-existing microcracks [8,9,10,11,12]. Merrian et al. [13] demonstrated a definite relationship between tensile strength and quartz content of the granite rocks they investigated. Irfan and Dearman [6] proposed a micropetrographic index, which is the ratio of sound to unsound constituents, to assess the grade of weathering of granite. They found a good relationship between the mechanical properties and the micropetrographic index of granitic rocks. Based on the analysis of micropetrographic data, Mendes et al. [14] also demonstrated that the mechanical properties of granites have a good correlation with their petrographic characteristics. Except for mineral composition, several investigations into the impact of grain size on the mechanical properties of rocks have been conducted. Brace [15] found that finer-grained rocks have higher mechanical strength. Onodera and Asoka Kumara [7] presented a linear relationship between grain size and strength of igneous rocks, that is, the strength increased as the grain size of granite decreased. Tugrul and Zarif [8] conducted a series of petrographic analyses and mechanical tests on granitic rock samples from different parts of Turkey. They argued that the mechanical properties of granites are linearly correlated with quartz and feldspar content, respectively, positively and negatively. However, this conclusion is in direct contrast to the work by Yilmaz et al. [11], Sousa [16], and Sajid et al. [17]. Sousa [16] evaluated the mechanical behaviors of different Portuguese granites based on their petrographic characteristics, including grain size, quartz fissuration, mineral deterioration, and the contacts between quartz and other mineral groups. He proposed that uniaxial compressive strength is not positively related to the quartz content or quartz-feldspar ratio, but decreases with increasing quartz content.

In rock engineering, microcrack analysis is of fundamental importance, and since its inception, it is now known that the growth of microcracks considerably controls the mechanical behavior and failure of rocks [18,19,20,21]. In the laboratory, several direct methods have been used for the analysis of microcracks, including petrographic investigation of thin sections, surface observation [22], the X-ray image technique [23,24,25], and the acoustic emission technique [12]. The results of microcrack analysis demonstrate that rock failure under uniaxial compressive loading results from the growth, interaction, and coalescence of a large number of pre-existing and newly-generated intergranular and intragranular microcracks. Combining digital image analysis with fluorescent and polarized microscopy of thin sections, Rigopoulos et al. [22] studied the microcrack patterns of a troctolite and a diorite before and after a compressive test, and argued that the intragranular microcracks are the dominating crack type in loaded specimens, in terms of their total number and length. A similar conclusion has been reported in granite [26,27]. Moreover, the granite texture controls the tensile crack development under uniaxial tensile loading. Fujii et al. [28] investigated the mineral composition and surface topography of tensile fractures in Inada granite in Japan after direct uniaxial testing, and indicated that the mineral compositions were different at the failure surface, and most cracks were intragranular cracks, with 90% of cracks cutting across the mineral grains.

Progress has been made by previous researchers on the relationship between mechanical properties of granitic rock and their petrographic characteristics based on laboratory tests and observation by using statistical methods. However, further research is required before this problem is well understood. As mentioned above, the mechanical behaviors are related to the intragranular and intergranular microcrack growth, which is difficult to study in a laboratory setting. Although X-ray computerized tomography can be used to differentiate the microcrack phase and mineral phase, the mineral constituents are difficult to distinguish [24,25]. Alternatively, with the rapid development of computer science, numerical modeling has become an important research method. Numerical modeling has become an alternative approach to simulate rock failure processes on different scales [29].

In recent years, the discontinuous approach—including lattice method [30,31], discrete element method (DEM) [32], and other non-continuum-based methods—has been developed to investigate fracture growth in intact or jointed rock masses. The discontinuous approach is more convenient for modeling fracture initiation, propagation, intersection, and coalescence. Taking the petrographic characteristics of granitic rocks into account, the grain-based microstructure model has been adapted to simulate the mechanical properties and failure behaviors of granitic rocks. Chen et al. [30] proposed a novel volume-compensated lattice method, which overcomes the defect that the Poisson’s ratio in the traditional lattice model is fixed to 1/3 for plane stress and 1/4 for plane strain. Using this novel lattice method, Chen et al. [31] initially investigated the effects of grain boundary properties and mineral orientation on the mechanical behaviors of two-dimensional polycrystalline materials. Universal Distinct Element Code (UDEC) is a commercial code based on DEM. Lan et al. [33] applied the Voronoi tessellation technique to divide the intact rock specimen into a large number of polygonal Voronoi cells, which can mimic the main rock-forming minerals in UDEC. The grain-based model is able to simulate the microscale heterogeneity caused by elastic variation and contact stiffness anisotropy. It was found that grain shape, size, and distribution significantly affect the macroscopic mechanical and deformation behaviors, and the failure process of brittle rocks under uniaxial compressive loading. By using this method, Mohsen and Martin [34] found that the crack initiation stress was also controlled by the material heterogeneity. Similarly, another grain-based modeling approach in two-dimensional Particle Flow Code (PFC2D) was developed by Potyondy [35], which aimed at mimicking grain-based materials, such as granitic rocks. Different from the grain-based modeling approach in UDEC, the grains in PFC2D can be cut across when cracks grow. The grain-based modeling approach in PFC2D was applied in several studies [36,37,38,39,40,41,42].

With increasing use of nuclear energy, the safe disposal of high-level radioactive waste has become a hot issue in recent years. The international nuclear energy community is proposing to bury high-level radioactive waste, which is a significant risk to the environment and humankind, deep underground. Deep granitic rock, with low permeability, is a candidate host rock for the repository of this high-level radioactive waste. In this study, we combined laboratory testing with the grain-based modeling method in PFC2D to investigate the mechanical strength and quasi-brittle failure behavior of Alashan granite, which is a candidate host rock for the repository of high-level radioactive waste, under uniaxial compressive and Brazilian indirect tensile testing. After model calibration, the influence of the mineral grain size and distribution on the strength and patterns of microcracks of granitic rocks were then examined and discussed.

## 2. Laboratory UCS and BTS Testing

### 2.1. Sample Preparation and Strength Test Facility

**Figure 1:**
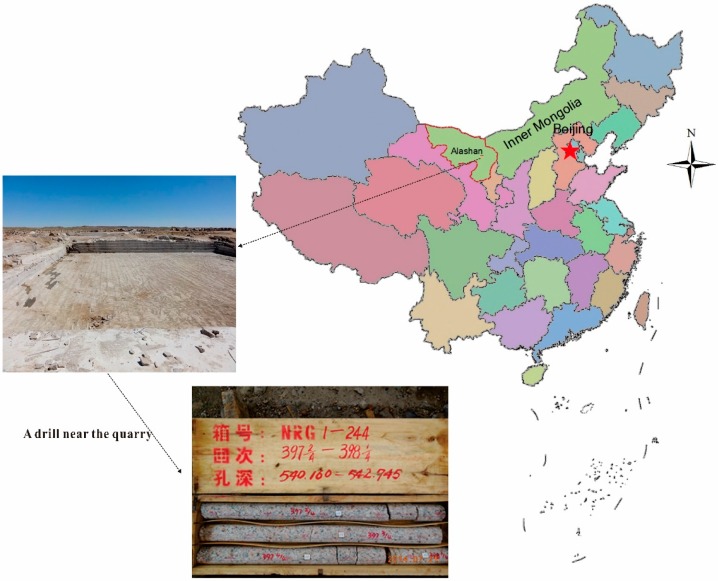
The location of the Alashan area and a photo of rock cores deeply drilled for test specimens.

**Figure 2:**
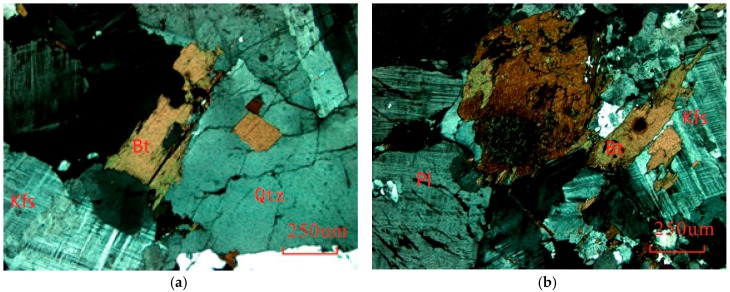
Two cross-polarized light photos of thin sections showing the mineralogical composition of test specimens. (**a**) Section I; (**b**) Section II.

The Alashan area, which is in the west of Inner Mongolia, China, is covered by a large area of granitic rock. According the geological survey results, Bayinnuorigong, in the Alashan area, may be a preferred candidate location, where the outcrop is a large area of porphyritic granite and granodiorite [43,44]. A deep drill (Figure 1) in a nearby open pit for building construction material revealed that, from the surface to 600 m underground, the rocks are porphyritic granite (0~417.5 m), granodiorite (417.5~517.3 m), and porphyritic granite (517.3~603.1 m). The test samples from drill cores with a diameter of 63 mm at 541 m are porphyritic granite with low water content and porosity. Based on the polarized microscopy observations, two typical photomicrographs of thin sections of the rocks were shown in Figure 2, which presents the microstructure and mineralogical composition. The rock is composed of approximately 25% quartz, 50% alkali feldspar, 15% plagioclase, and 10% other minerals (biotite and hornblende). The average grain sizes of quartz, alkali feldspar, and plagioclase varied from 0.8 to 4.0 mm, 4 to 9 mm, and 2 to 8 mm, respectively. The average density was 2650.3 kg/m^3^, and the average longitudinal wave and shear wave velocity were 5860.4 m/s and 3151.8 m/s, respectively. Several rock specimens were prepared for uniaxial compressive testing and Brazilian tensile testing according the ISRM Suggested Method [45,46].

**Figure 3:**
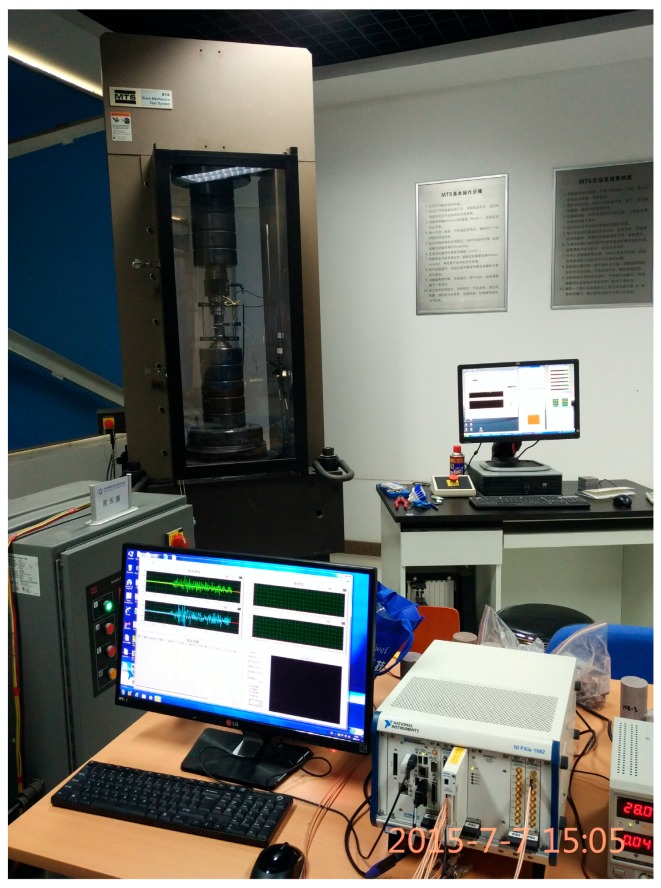
MTS 815 servo-controlled hydraulic testing machine for the laboratory tests.

The mechanical properties tests, including the uniaxial compressive strength test and uniaxial tensile strength by Brazilian indirect tensile test, were conducted using the MTS 815 servo-controlled hydraulic testing machine (Figure 3) at the Institute of Crustal Dynamics, China Earthquake Administration. The axial load was recorded by the sensor of an MTS Model 661.98C/D-03, and the axial displacement was recorded by a LVDT (Linear Variable Displacement Transformer). In addition, the long strain gauge of MTS Model 632.12E/F-20 was used for the measurement of lateral strain. During the UCS testing, the loading was controlled by axial displacement at a rate of 0.06 mm/min, while the rate was 0.03 mm/min under the UTS test.

### 2.2. Experimental Results

**Figure 4:**
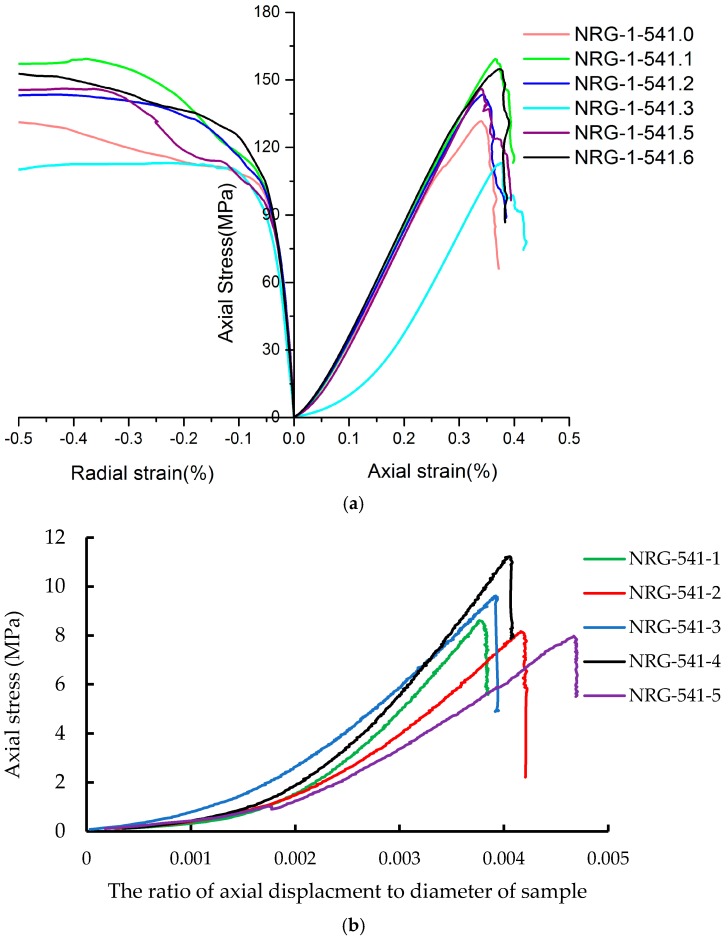
The curves of axial stress versus deformation of different specimens under UCS and UTS testing. (**a**) The stress–strain curves under UCS testing; (**b**) The curves of axial stress versus axial deformation under UTS testing.

**Figure 5:**
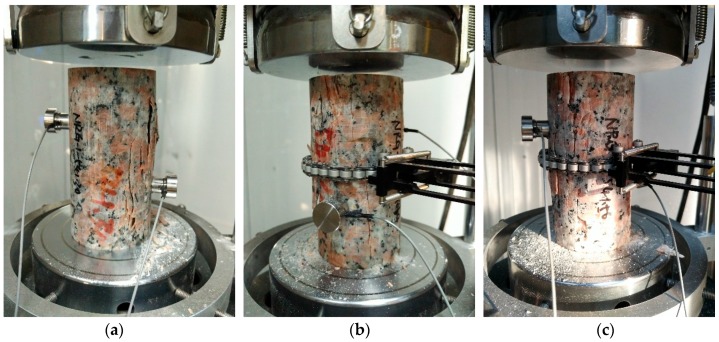

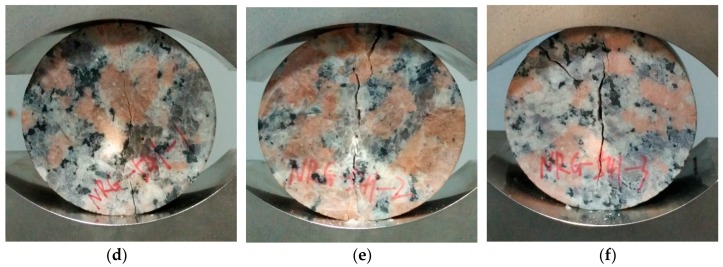
The failure characteristics of the rock specimens under both UCS and UTS testing. (**a**) NRG-1-541.3; (**b**) NRG-1-541.4; (**c**) NRG-1-541.5; (**d**) NRG-541-1; (**e**) NRG-541-2; (**f**) NRG-541-3.

Figure 4 shows the stress–strain curves of the porphyritic granite samples under UCS and UTS testing. Figure 4a shows that the UCS of the tested samples varied from 113.1 to 159.3 MPa, and the axial strains at peak strength were between 0.35% and 0.40%. The average elastic modulus and Poisson’s ratio were 49.5 GPa and 0.24, which were calculated according the linear segment of stress–strain curves except for the sample of NRG-1-5413. Figure 4b presents the curves of axial stress versus axial deformation under indirect Brazilian testing, and the UTS values range from 7.8 to 11.0 MPa. As aforementioned in the literature review, the strength of porphyritic granite is controlled by petrographic texture, which controls the microcrack initiation, propagation, and coalescence. In our experiments, no X-ray tomography technique has been applied. The process of microcrack growth in rock samples cannot be observed and predicted. However, from the observation of the samples’ surface, it was found that the rock specimens’ failure was caused by many split cracks under both UCS and UTS testing (Figure 5). Most of the observed cracks cut across the rock-forming minerals, such as alkali-feldspars, quartzes, and plagioclase. The microcracking characteristics will be studied in the following sections from the view of numerical modeling by the grain-based model in PFC2D.

## 3. Numerical Modeling Methodology

### 3.1. Particle Flow Code

**Figure 6:**
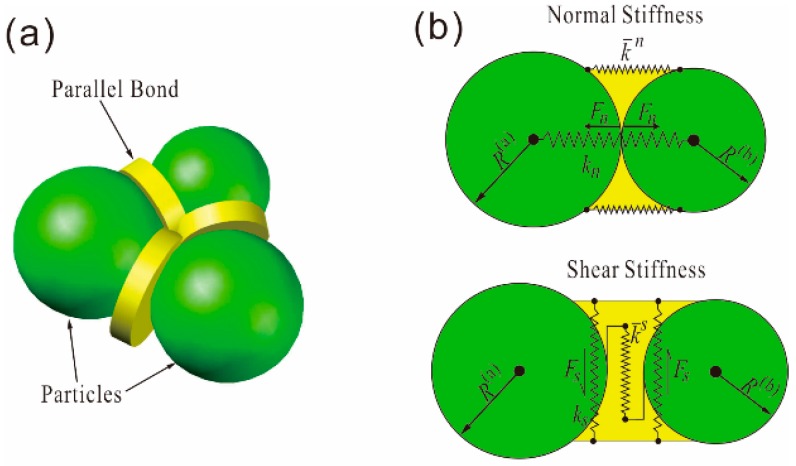

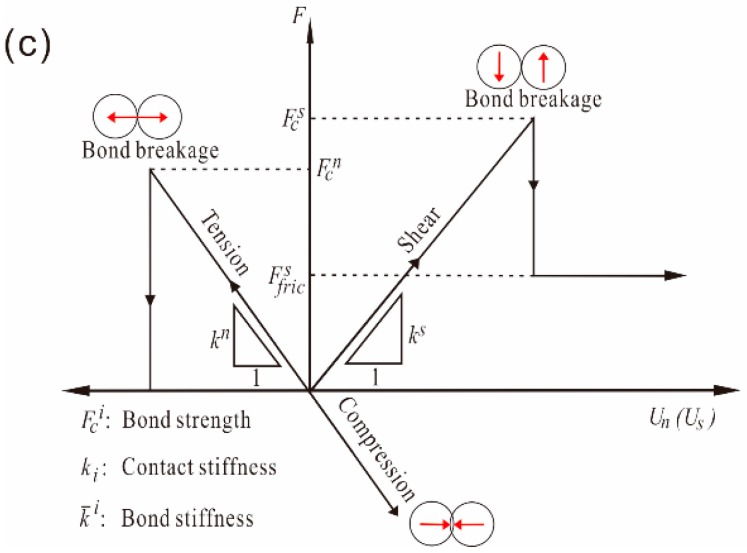
Bonded particle model in PFC2D: (**a**) Schematic of bonded particle model; (**b**) the micro-scale parameters of the bond at contacts; and (**c**) the deformation and failure mechanisms of the bond under tensile and shear stress (modified from Bahaaddini et al. [48]).

PFC2D is a distinct element modeling method in which the solid materials are represented as an assembly of circular particles. Although PFC2D is based on the discontinuum method, with the help of bond models at the contacts between round particles, it can also be used to model the deformation behavior of the continuum. With the properties of normal and shear stiffness, as well as shear and tensile strength, the bonds can simulate deformation and microcrack development based on the relationship presented by Potyondy and Cundall [47]. In rock mechanics research, the parallel bond model is among the most frequently used models. The corresponding microscale properties, and deformation and failure behaviors, are presented in Figure 6. Normally, the Young’s modulus (*E)* of an emulated rock sample is related to the specified contact micro-stiffness. The Poisson’s ratio (*ν*) is affected by the ratio of normal to shear stiffness. The microscale parameters in this method are different from the macroscale parameters, such as *E* and *ν*, which can be directly measured in the laboratory. These parameters have to be calibrated according to laboratory results from the confined/unconfined compressive test and the direct tensile test. Generally, by adjusting the micro-stiffness and micro-strength of particles, a realistic rock can be reproduced. Under the applied load, when the maximum shear stress or tensile stress acting on the bond exceeds the shear or tensile strength, the bond will break in shear or tensile mode, resulting in shear or tensile microcracks, respectively. With the ongoing generation of microcracks, a macro-fracture can be formed by the linking of these individual microcracks.

### 3.2. Grain-Based Model

**Figure 7:**
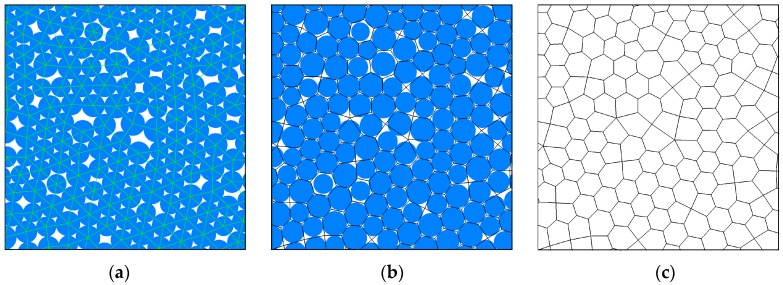

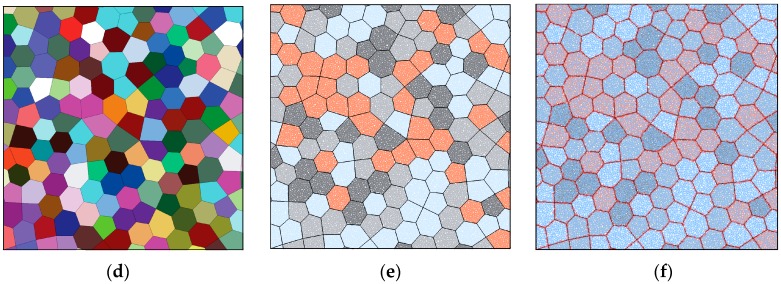
Steps in the generation of a grain-based model based on the initial particle seeds in PFC5.0 (**a**–**f**). (**a**) Initial particle packing; (**b**) Development of polygons; (**c**) Polygonal grain structure; (**d**) Different grain types; (**e**) Particles in mineral grians bonded by parallel bond; (**f**) Grain boundaries mimiced by smooth joint model (red).

The grain-based model (GBM) in PFC2D was first developed by Potyondy [35], who applied this method to investigate the influence of heterogeneous material on rock strength and microcrack development. According to the grain-based model, the petrographic texture in real rocks can be well described, including the mineral constituents and grain size and distribution. Potyondy [35] and Bahrani and Kaiser [40] have described the details of the procedure to generate a grain-based model in PFC2D4.0. After recoding this algorithm in PFC5.0 [49], Figure 7 presents an example of generating a grain-based model, Firstly, an initial circular particle packing model was developed (Figure 7a), where the particle size was calculated according grain size distribution. Then, a polygonal mineral structure was generated by the algorithm used by Potyondy [35], and each polygon was filled by a different color representing different mineral grains (Figure 7b–d). Thirdly, a base bonded circular particle model with a smaller diameter was overlaid by the mineral structure, and the contacts and particle properties were modified to correspond with the real minerals (Figure 7e). Lastly, the circular particles in the grain interface were assigned with smooth joint contact, and in grains the parallel bonded contact model was used (Figure 7f).

### 3.3. Smooth Joint Model

The general method used to emulate natural joints in PFC is the bond removal method, in which the particle contacts lying on a joint track are left unbonded. This approach has been used to study the shear behavior of rock joints in a number of studies [50,51,52]. According to Bahaaddini et al. [48], the ability of the bond removal method to reproduce the shear behavior of rock joints is limited because of the circular shape, unequal size of the particles, and non-uniform distribution, leading to an unrealistic shear behavior of rock joints. In order to overcome the shortcomings of this approach, Pierce et al. [53] introduced the Smooth Joint Model (SJM) into PFC.

The SJM emulates the behavior of joints by micro-scale slip surfaces at the contacts between particles that lie opposite to each other at the joint interface, as shown in Figure 2a. At these contacts, the parallel bonds are removed and the new bonding model is assigned with pre-defined orientation, whereas contacting particles can overlap and pass through each other [53]. The details of the fundamental algorithm of the SJM can be acquired from the software manuals [49], while a summary of this model in PFC2D code is given here.

**Figure 8:**
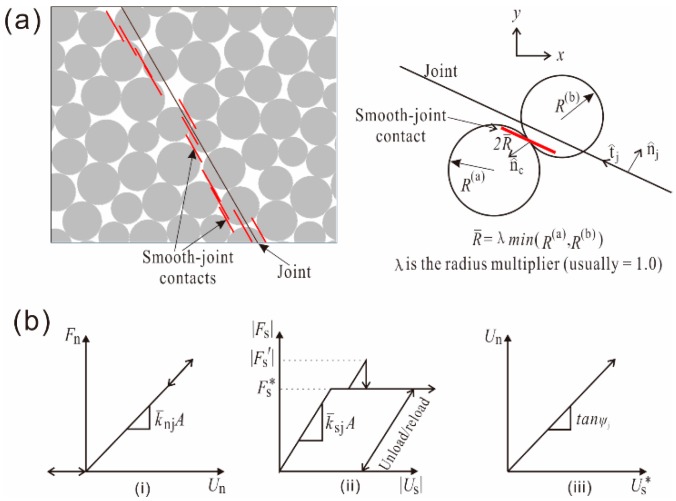
Smooth joint model [49]: (**a**) Sketches of smooth joint model; (**b**) force-displacement relationship at smooth joint contacts: (i) normal force versus normal displacement; (ii) shear force versus shear displacement; and (iii) normal displacement versus shear displacement in the sliding process.

In SJM, the force **F** and relative displacement **U** at a contact point can be given as

(1) $$F\;=\;F_{n}{\hat{n}}_{j}+F_{s}$$

(2) $$U\;=\;U_{n}{\hat{n}}_{j}+U_{s}$$

where ${\hat{n}}_{j}$ is the normal unit vector at contact as shown in Figure 8a. The positive values of *F_n_* and *U_n_* represent the compression force and overlapping of particles, respectively, while **F_s_** and **U_s_** represent the shear force vector and shear displacement vector, respectively. The force–displacement relationship of each smooth joint contact point follows the Coulomb sliding model with dilation, as shown in Figure 8b. Micro-scale parameters—such as SJM normal stiffness ${\bar{k}}_{nj}$, SJM shear stiffness ${\bar{k}}_{sj}$, SJM friction coefficient $\mu_{j}$, and SJM dilation angle $\varphi_{j}$—reflect the mechanical behavior of a smooth joint contact with the area of cross section *A*.

The increments of joint force are calculated by the elastic components of the displacement increment multiplying the SJM normal and shear stiffness. The normal force and shear force are updated as

(3) $$F_{n}:=F_{n}+{\bar{k}}_{nj}A\;\Delta \;U_{n}$$

(4) $${F_{s}}^{\prime }:=F_{s}-{\bar{k}}_{sj}A\;\Delta \;U_{s}$$

If $\left|{F_{s}}^{\prime }\right|\leq (F_{s}^{*}=\mu_{j}F_{n})$, then $\left|F_{s}\right|=\left|{F_{s}}^{\prime }\right|$. If $\left|{F_{s}}^{\prime }\right|>F_{s}^{*}$, then sliding occurs at the smooth joint contact and shear force and normal force are updated as

(5) $$\left|F_{s}\right|=F_{s}^{*}$$

(6) $$F_{n}:=F_{n}+[\Delta U_{s}^{*}\tan\varphi_{j}]{\bar{k}}_{nj}A=F_{n}+(\frac{\left|{F_{s}}^{\prime }\right|-F_{s}^{*}}{{\bar{k}}_{sj}}){\bar{k}}_{nj}\tan\varphi_{j}$$

## 4. Parameter Calibration of Grain-Based Model for Porphyritic Granite

The parameters used in PFC2D for simulating porphyritic granite samples are microscale parameters that cannot be directly acquired from experimental laboratory tests. Parameter calibration is a critical step in numerical modeling by PFC2D. Generally, it is a trial and error process to calibrate microparameters based on a series of UCS and UTS tests. Fortunately, the relationship between microparameters and macroparameters have been investigated in several prior studies [47,54], which can serve as guidance for the optimization of the microparameters for a specific rock. In these experiments, the elastic modulus of the model is controlled by the stiffness of the contact, and the Poisson’s ratio is decided by the ratio of normal stiffness to shear stiffness at the contact. The microscale tensile strength, cohesion, and friction angle affect the sample strength.

The model used in parameter calibration has a height of 80 mm and a width of 40 mm, which was assembled by about 55,560 circular particles with radii ranging from 0.1 to 0.155 mm. The average mineral grain size is assumed to be 4 mm, although the size of some minerals in real rock reach 8 mm or more. The minerals are randomly distributed in the numerical rock model and the volume contents are 50%, 25%, 15% and 10% for alkali feldspar, quartz, plagioclase and biotite and others, respectively. The macro elastic parameters and strength are comparable with the mineral properties suggested by previous studies [38,42]. In all of the minerals, the strength, elastic modulus, cohesion, and frictional angle are the highest, and biotite had the highest density. All the smooth joint contacts at the grain boundary were simplified to the same one and were assigned with the same set of microparameters. Lastly, after a series of trial and error, the optimized microparameters—which can be used to reproduce the macroscale strength and deformation behaviors of the porphyritic granite samples—were established, as listed in Table 1.

**Figure 9:**
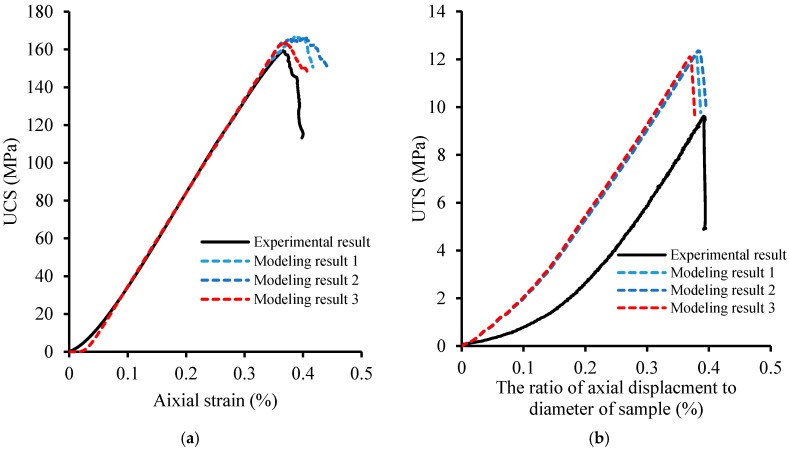
Microscale parameter calibration for Alasha granite by modeling of UCS and UTS tests. (**a**) The stress–strain curves under UCS testing; (**b**) The curves of axial stress vs. axial deformation under UTS testing.

**Figure 10:**
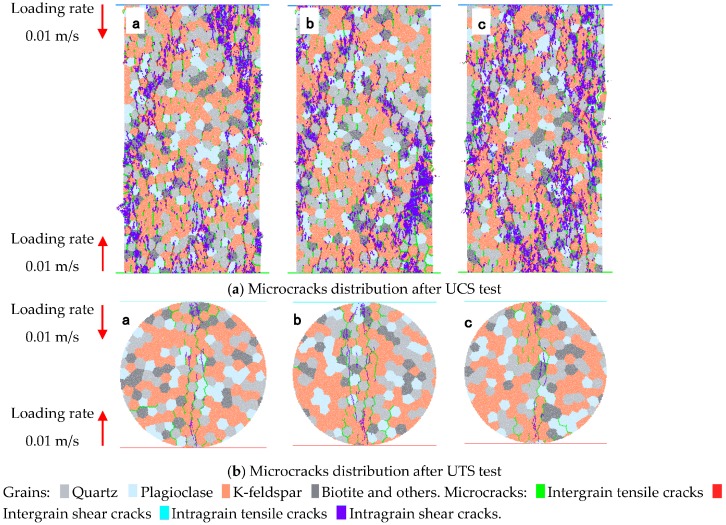
Mineral distributions and microcrack distributions in rock models after UCS and UTS tests under the loading rate of 0.01 m/s. a, b, c are the indexes of samples, respectively. (**a**) Microcracks distribution after UCS test; (**b**) Microcracks distribution after UTS test.

According the characteristics described in the last paragraph, three models with random distribution minerals were generated. Figure 9 presents stress–strain curves of the numerical models in the uniaxial compressive test and indirect tensile test, from which the average UCS, UTS, and elastic modulus were obtained as 164.6, 12.2, and 49.7 GPa, respectively. The UCS and elastic modulus in modeling agree with the corresponding values in laboratory experiments. Although the UTS is a little higher than the experimental result, the ratio of UCS to UTS reaches 13.7, which is within a reasonable range [55,56]. The microcrack patterns in rock models, after UCS and UTS tests (Figure 10), show many intragranular microcracks, which agree with the laboratory experimental results.

The previous laboratory experimental research [11,17,18] shows that grain size is one of most important factors influencing the strength of granitic rocks. In the following section, a series of numerical simulations of the models under uniaxial compressive loading and Brazilian indirect tensile testing were conducted using the microparameters listed in Table 1. The influence of grain size on microcrack development and strength of granite will be analyzed and discussed.

## 5. Effects of Grain Scale on Granite Strength

### 5.1. Model Descriptions

**Figure 11:**
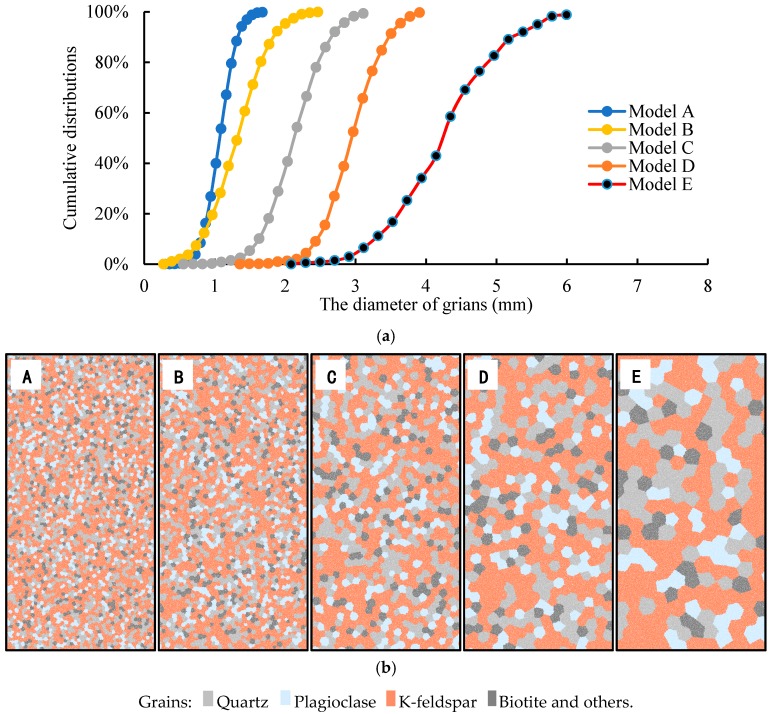
Cumulative distributions of the size of mineral grains in the five models and the corresponding mineral structures. (**a**) Cumulative distributions of the size of mineral grains in the five models; (**b**) Mineral structures in five different models.

The effects of grain scale on granite strength and microcrack growth will be discussed in this section. Five series models (A–E) were generated with different mineral distributions, of which the average grain diameters were about 1.0, 1.2, 2.0, 2.8, and 4.0 mm, respectively (Figure 11a). The grain size of model A is the most uniform, while model E has the maximum variety coefficient of grain size. The particles number in each grain is generally determined by the ratio of average diameter of particles to average grain size. In each set model, from A to E, the number of particles for the smallest grain are 3, 3, 18, 16, and 93, respectively. Figure 11b shows the mineral structures of the models. In these models, except for grain size and distribution, all the other macroscale physical parameters and microparameters are constant as in Section 4. The brown-colored grains are alkali-feldspar, the light gray grains mimic quartz, the plagioclose are represented by hoary-colored grains, and the deep gray grains represent biotite and other black minerals.

### 5.2. Numerical Strength Testing Results

**Figure 12:**
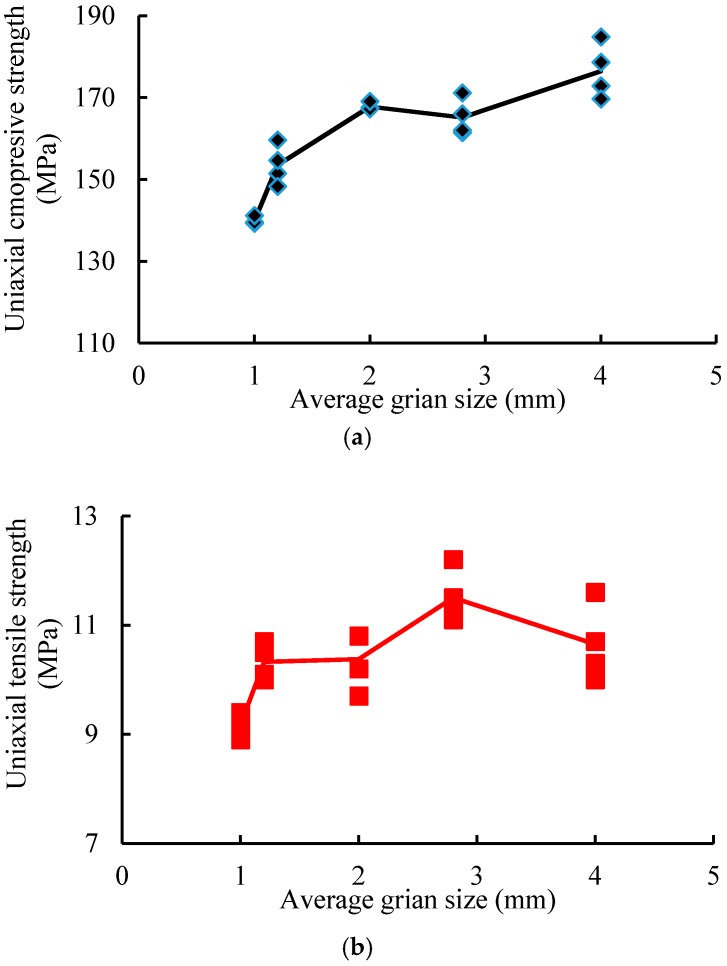

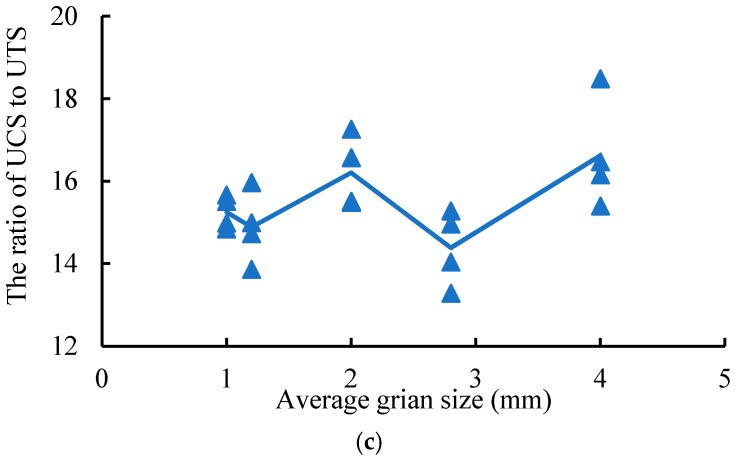
UCS, UTS, and the ratio of UCS to UTS varying with average grain size, respectively. (**a**) UCS varying with average grain size; (**b**) The UTS varying with average grain size; (**c**) The ratio of UCS to UTS varying with average grain size.

After a series of numerical simulations, the uniaxial compressive strength and Brazilian indirect tensile strength of the numerical models with different average grain sizes are summarized in Table 2. Obviously, the grain size has a great effect on rock strength. The average UCS values for model A to model E are 139.8, 153.4, 167.8, 165.1, and 176.5 MPa; and the average UTS values are 9.2, 10.3, 10.4, 11.5, and 10.7 MPa. So, the average ratio of USC to UTS varies from 14.4 to 16.6, which agrees with the statistical results of Cai [56]. Figure 12 shows the UCS, UTS, and the ratio of UCS to UTS varying with the average grain size of the models. We found that the UCS increases with increasing average grain size. Model A, with the smallest average grain size, had the lowest UCS, while model E, with the largest average grain size, had the highest UCS. However, this observation is the opposite of the laboratory experimental results. The UTS also increases with increasing average grain size, except in model E. Although the mineral content is constant in each series model, the UCS and UTS of the models are affected by mineral distributions.

### 5.3. Patterns of Microcracks

**Figure 13:**
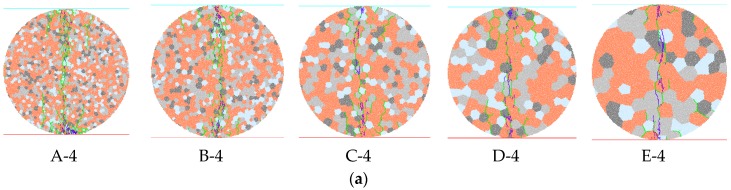

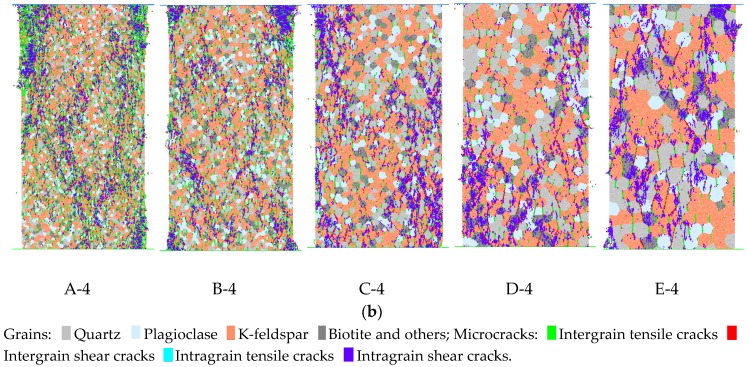
Mineral distributions and microcrack distributions in five representative rock models after UCS and UTS tests. (**a**) Micro-cracks distribution after UTS tests; (**b**) Micro-cracks distribution after UCS tests.

**Figure 14:**
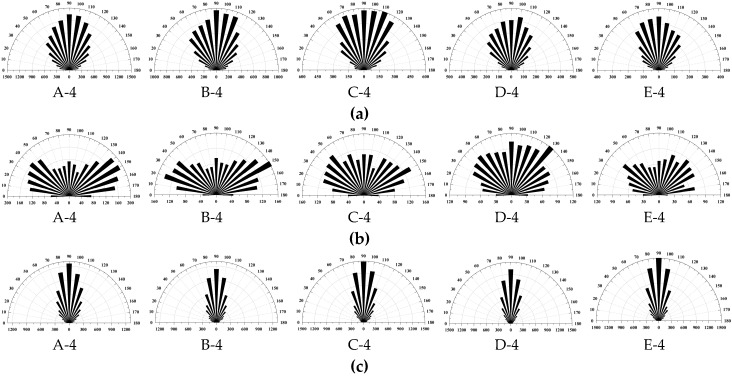
The rose diagrams of the inclination angle of different type microcracks in the corresponding models in Figure 13b. (**a**) The rose diagram of inclination angle of intergrain tensile microcracks; (**b**) The rose diagram of inclination angle of intergrain shear microcracks; (**c**) The rose diagram of inclination angle of intragrain tensile microcracks.

Microcracks develop when rock samples fail under loading. Previous studies have shown that the number of microcracks will gradually increase with increased loading [12,15]. In the present study, taking a model from each series, the microcrack patterns, after rock sample failure, are presented in Figure 13, where the tensile and shear types of microcracks between or in mineral grains were included. The summary of the generated microcrack types are listed in Table 3. We found that most of the generated microcracks were tensile cracks, both after UTS and UCS tests. Under UTS tests, most of the generated microcracks were intergrain tensile cracks, and the second-most common microcracks were intragrain; with increasing the average grain size, the number of intragrain microcracks increased, while the number of intergrain microcracks decreased. This indicates more cracks are generated in mineral grains if the average grain size is larger. Under uniaxial loading, most of the generated microcracks in models A and B were intergrain tensile cracks, while in models C–E, the most common microcracks were intragrain tensile cracks. This means intergrain tensile cracks are more easily induced in the model with low average grain size. When increasing the average grain size in numerical models, the ratio of the number of intragrain tensile cracks to the number of intergrain tensile cracks also increased from 0.6 to 2.5. Comparing this ratio with the UCS, we conclude that the UCS of the rock samples is positively correlated with the ratio of the number of intragrain tensile cracks to the number of intergrain tensile cracks. Based on the statistical data of inclination angle of the microcracks between the microcracking direction and the horizontal direction in an anticlockwise direction, Figure 14 presents the rose diagrams of inclination angle of three types of microcracks, including intergrain tensile microcracks, intergrain shear microcracks, and intragrain tensile microcracks. Most of the intergrain and intragrain tensile microcracks were inclined at about 70° to 110°, while the intergrain shear microcracks were inclined at about 10° to 40° and 140° to 170°. However, the variation of grain size distribution does not have a significant influence on the inclination of generated microcracks.

### 5.4. Discussion

Laboratory experimental results presented in this paper show that the UCS of Alashan granite varies from 113.1 MPa to 159.3 MPa, and the UTS values, based on Brazilian indirect tensile testing, range from 7.8 MPa to 11.0 MPa. Meanwhile, it is possible to calibrate the UCS, UTS, and elastic properties of Alashan granite using the grain-based modeling approach. The modeling results of the ratio of UCS to UTS are in the reasonable range of 10 to 20. During the modeling, in order to match the UCS and UTS at the same time, the smooth joint normal strength (tensile strength of mineral boundary) needs to be a low value.

In physical experiments, the UCS and UTS all decrease with increasing mineral diameter [8,11,18]. Eberhardt et al. [12] pointed out that the longer grain boundaries, due to larger grain size, provided more continuous paths of weakness for growing cracks to propagate along, and the rock strength decreased with increasing grain size, not by earlier crack initiation, as dictated by Griffith’s theory, but through a process where cracks propagate more easily along longer existing weakness planes. However, the opposite result—that rock strength is positively correlated with grain size—is presented in our modeling. Hofmann [38] pointed out that in numerical modeling, smaller minerals are bonded by fewer particles resulting in a higher stress concentration on these few particles, leading to reduction of the rock strength. However the authors do not agree with this interpretation. From the observation of the development of microscale fractures in our modeling, a phenomenon has been found that the direction of fracture propagation is more easily reoriented in fine grained model than in coarse grained model, which is helpful for fracture propagation in preferred direction. In real granitic rock, some coarse minerals are cut by the cleavage planes, which results in the mechanical behaviors of coarse grained granitic rock being the same as those of fine granitic rock. Thus, the authors hypothesize the discrepancy could be related to the modeling of cleavage minerals, such as plagioclase or alkali-feldspar. Future studies should be aimed at further investigation into this effect.

From direct observation of the samples’ surface, it was found that the minerals in the granite samples were cut across by cracks under both UCS and UTS tests. The grain-based approach in PFC2D accurately mimicked the process of minerals being cut by microcracks, while the minerals cannot be cut in the grain-based approach in UDEC [33]. Laboratory observations of the thin section of the rock samples, including tensile fracture, have shown that most of the fractures are intragranular cracks, with 90% of the fracture cutting across the mineral grains [28]. Rigopoulas et al. [22] also argued that the intragranular microcracks are the dominant crack type. In our modeling, most of the generated microcracks were tensile-type cracks, including intergrain and intragrain cracks, after UCS and UTS tests, which basically agrees with the experimental observation. We also found a new correlation where the average mineral size influences the ratio of intragrain to intergrain microcracks.

## 6. Conclusions

Laboratory experiments and numerical simulations are both important approaches for researching the brittle failure process in rocks. In this paper, the mechanical strengths and elastic properties of coarse-grained porphyritic granite at 541 m underground, in the Bayinnuorigong area of Alashan, were obtained by the uniaxial compressive test and Brazilian indirect tensile test. After that, the grain-based approach in PFC2D was introduced and applied to investigate the microcracking behaviors of granitic samples, and the grain scale effect was analyzed. The main results are summarized as follows:

(1) Using the grain-based approach in PFC2D, it is possible to reproduce the UCS and UTS of Alashan granite. Based on the macroscale mechanical properties of Alashan granite, a successful and valid calibration can be achieved using a model with a height of 8 cm and a width of 4 cm. The modeling results of the ratio of UCS to UTS are in a reasonable range of 10 to 20.
(2) The numerical simulation reveals that the mineral size has a significant influence on the simulated rock strength, and a positive relationship was found between UCS, UTS, and the average mineral diameter in the modeling. This finding may be related to the phenomenon that the direction of fracture propagation is more easily reoriented in the fine grained model than in coarse grained model.
(3) Most of the generated microcracks are tensile cracks both after the UTS and UCS tests. Under the UTS test, with increased average grain size, the number of intragrain microcracks also increases, while the number of intergrain cracks decreases; under uniaxial loading, the ratio of the number of intragrain tensile cracks to the number of intergrain tensile cracks increases as the average grain size increases. The UCS of numerical rock samples is positively related to the ratio of the number of intragrain tensile cracks to the number of intergrain tensile cracks. The variation in grain size distribution has no or minimal influence on the inclination of induced microcracks.

To summarize, the grain-based approach in PFC2D has the ability to mimic the mechanical strength and microcrack development process in granitic rocks under loading. However, there are some defects in the modeling. Firstly, the UCS and UTS of samples are positively related to the average mineral diameter in the modeling, which is not supported by the results in the physical experiments; secondly, the different parameters of the smooth joint contacts for the boundaries of different minerals should be considered; and thirdly, three-dimensional petrographic texture of real granite samples should also be investigated. These topics should be addressed in future studies.

## Figures and Tables

**Table 1:** Input microscale parameters for modeling of cement mortar and natural fractures.

Input Microparameters	Values			
	Quartz	Alkila-Feldspar	Plagioclase	Biotite
Mmicroparameters for minerals				
Minimum particle radius, *R*_min_ (mm)	0.1	0.1	0.1	0.1
Ratio of particle radius, *R*_max_/*R*_min_	1.55	1.55	1.55	1.55
Particle density, *ρ* (kg/m^3^)	2600	2650	2650	2850
Modulus of the particle, *E*_c_ (GPa)	60	45	40	23
Contact normal to shear stiffness ratio, *k*_n_/*k*_s_	1	2	2	3
Friction coefficient of particle, *μ*	0.8	0.8	0.8	0.8
Parallel bond Modulus, ${\bar{E}}_{c}$ (GPa)	60	45	40	23
Parallel bond normal to shear stiffness ratio, ${\bar{k}}_{n}/{\bar{k}}_{s}$	1	2	2	3
Parallel bond tensile strength, ${\bar{\sigma }}_{t}$ (MPa)	50	45	45	40
Parallel bond shear strength, $\bar{c}$ (MPa)	90	113	113	125
Parallel bond friction angle, $\phi^{\prime }$ (°)	50	48	48	45
Parallel bond radius multiplier, $\bar{\lambda }$	1	1	1	1
Moment contribution factor, β	0.2	0.2	0.2	0.2
Mmicroparameters for mineral boundaries (Smooth joint contact)				
SJM bond normal stiffness factor	0.5			
SJM bond shear stiffness factor	0.8			
SJM bond tensile strength, ${{\bar{\sigma }}_{t}}^{\prime }$ (MPa)	10			
SJM bond cohesion, ${\bar{c}}^{\prime }$ (MPa)	60			
SJM bond friction angle, $\phi^{\prime }$ (°)	40			
SJM bond friction coefficient, *μ’*	0.8			

**Table 2:** The summary of uniaxial compressive strength and Brazilian indirect tensile strength.

Sample	Average Grainsize (mm)	Uniaxial Compressive Strength (MPa)	Brazilian Tensile Strength (MPa)
A-1	1.0	139.4	8.9
A-2	1.0	139.3	9.3
A-3	1.0	139.5	9.4
A-4	1.0	141.1	9.1
B-1	1.2	151.4	10.1
B-2	1.2	154.6	10.5
B-3	1.2	159.6	10.0
B-4	1.2	148.3	10.7
C-1	2.0	167.5	10.8
C-2	2.0	167.4	9.7
C-3	2.0	167.2	10.8
C-4	2.0	169.0	10.2
D-1	2.8	161.4	11.5
D-2	2.8	166.0	11.1
D-3	2.8	162.0	12.2
D-4	2.8	171.1	11.2
E-1	4.0	172.8	10.7
E-2	4.0	184.8	10.0
E-3	4.0	178.6	11.6
E-4	4.0	169.6	10.3

**Table 3:** The summary of microcrack information in rock samples after UCS and UTS tests.

Sample	Test Modeling	Number of Different Types of Microcracks			
		Intragrain Shear Cracks	Intragrain Tensile Cracks	Intergrain Shear Cracks	Intergrain Tensile Cracks
A-4	BTT	2	315	40	1503
B-4	BTT	1	301	29	1168
C-4	BTT	0	270	21	1053
D-4	BTT	6	331	11	1011
E-4	BTT	3	385	36	620
A-4	UCT	51	7977	2420	13,219
B-4	UCT	68	6994	1922	9331
C-4	UCT	133	8485	1910	5998
D-4	UCT	74	6993	1560	4085
E-4	UCT	174	8252	1237	3298
